# Fast Disintegrating Combination Tablet of Taste Masked Levocetrizine Dihydrochloride and Montelukast Sodium: Formulation Design, Development, and Characterization

**DOI:** 10.1155/2014/568320

**Published:** 2014-03-30

**Authors:** M. M. Gupta, Niraj Gupta, Bhupendra S. Chauhan, Shweta Pandey

**Affiliations:** ^1^School of Pharmacy, Faculty of Medical Sciences, The University of the West Indies, St. Augustine, Trinidad and Tobago; ^2^Department of Pharmaceutics, Jaipur College of Pharmacy, Sitapura, Tonk Road, Jaipur, Rajasthan 302022, India

## Abstract

The aim of this study was to prepare fast disintegrating combination tablet of taste masked Levocetrizine dihydrochloride and Montelukast sodium by using direct compression method. To prevent bitter taste and unacceptable odour of the Levocetrizine dihydrochloride drug, the drug was taste masked with ion exchange resins like Kyron-T-104 and Tulsion-412. Among the two resins, Kyron-T-104 was selected for further studies because of high drug loading capacity, low cost, and better drug release profile. An ion exchange resin complex was prepared by the batch technique and various parameters; namely, resin activation, drug: resin ratio, pH, temperature, and stirring time, and swelling time were optimized to successfully formulate the tasteless drug resin complex (DRC). The tablets were prepared using microcrystalline cellulose (MCC) PH 102 as diluent along with crospovidone (CP), croscarmellose sodium (CCM), and sodium starch glycolate (SSG) as a superdisintegrants. The tablets were evaluated for weight variation, hardness, friability, wetting time, water absorption ratio, disintegration time (DT), and dissolution study and it was concluded that the tablet formulation prepared with 2% SSG + CCS showed better disintegration time in comparison with other formulation and good drug release. The stability studies were carried out for the optimized batch for three months and it showed acceptable results.

## 1. Introduction

Various physiological and neurological conditions like dysphagia, motion sickness, and hand tremors lead to noncompliance of conventional oral dosage forms. Mouth dissolving drug delivery systems (MDDDS), orally disintegrating system (ODT), and fast disintegrating tablet (FDT) are especially designed for dysphagic, geriatric, pediatric, bed-ridden, travelling, and psychotic patients who are unable to swallow or refuse to swallow conventional oral formulations. As they dissolve/disintegrate very fast when placed in the mouth, FDT are the most convenient dosage forms for dysphagic, pediatric, and geriatric patients with swallowing problem [1–3]. They do not require water for administration and thus are a good alternative for travelers and for bed ridden patients. They simply vanish when placed in the mouth and so cannot be hidden in mouth by psychotic patients. These products not only increase the patient's compliance but also fetch large revenues to manufacturers due to line extension of the existing formulation [4–6].

FDT or MDDDS display a fast and spontaneous deaggregation in the mouth, soon after it comes in contact with saliva, dissolving the active ingredient and allowing absorption through all possible membranes it comes in contact with during deglutition [7–9].

Recently, several new advanced technologies lyophilization, moulding, direct compression, cotton candy process, spray drying, sublimation, mass extrusion, nanonization, and quick dissolve film formation have been introduced for the formulation of mouth dissolving tablets (MDTs) or fast disintegrating system with very interesting features, like extremely low disintegration time, exceptional taste masking ability, pleasant mouth feel, and sugar free tablets for diabetic patients [10, 11]. These techniques are based on the principles of increasing porosity and/or addition of superdisintegrants and water soluble excipients in the tablets [12–14].

Ion exchange resins have been increasingly used for the taste masking of bitter taste drugs and help to prepare fast disintegrating tablets. Thus, the taste masking of bitter active substances is a critical hurdle to overcome for the successful development of oral formulations [15–17]. Levocetrizine dihydrochloride is an orally active and R-enantiomer of cetrizine and is a third generation, nonsedating selective peripheral H1-receptor antagonist used in seasonal allergic rhinitis, perennial allergic rhinitis, and chronic urticaria [18, 19]. Allergy is a common problem among all age groups. Levocetrizine dihydrochloride is rapidly absorbed after oral administration and half-life is 8.3 hr which makes it suitable for once a day formulation. These diseases require rapid onset of action in order to provide fast relief. Unfortunately, it is accompanied with a very unpleasant bitter taste so it requires taste masking [20–22].

Montelukast sodium is a leukotriene receptor antagonist (LTRA) used in maintenance treatment of asthma and to relieve symptoms of seasonal allergies. It is usually administered orally [23–25]. In the present study an attempt had been made to prepare fast disintegrating combination tablets of Montelukast sodium and taste masked Levocetrizine dihydrochloride for the treatment of allergic rhinitis using coprocessed superdisintegrants containing crospovidone, croscarmellose sodium, and sodium starch glycolate. The coprocessed superdisintegrants help to increase the water uptake with shortest wetting time and thereby decrease the disintegration time of the tablets. These systems may offer superior profile with potential mucosal absorption, thus increasing the drug bioavailability. These systems are also called mouth dissolving tablets, melt-in-mouth tablets, reprimelts, porous tablets, orodispersible, quick dissolving, or rapidly disintegrating tablets.

## 2. Materials and Methods

### 2.1. Materials

Montelukast sodium, sodium starch glycolate, croscarmellose sodium, and crospovidone were procured as gift sample from MMC Health care pvt., Ltd., Baddi, India. Levocetrizine dihydrochloride was a generous gift from Amol Pharmaceuticals, Jaipur, India. Tulsion-412 and Kyron-T-104 were obtained as gift samples from Cadila Pharmaceuticals, Ahemdabad, India. All other materials (microcrystalline cellulose PH 102, Magneshium stearate and Talc) and chemicals used were of analytical reagent grade.

### 2.2. Analysis of Levocetrizine Dihydrochloride and Montelukast Sodium

The solution containing 20 *μ*g/mL of Levocetrizine dihydrochloride and Montelukast sodium in phosphate buffer (pH 6.8) was prepared and scanned over range of 200–400 nm against phosphate buffer (pH 6.8) as a blank using double beam UV spectrophotometer. The maximum wavelength was found to be 231.0 nm and 352.20 nm for Levocetrizine dihydrochloride and Montelukast sodium, respectively, which confirmed to the reported value.

### 2.3. Formulation of Drug (Levocetrizine Dihydrochloride): Resin Complex

Formulation of drug resin complex (DRC) of Levocetrizine dihydrochloride was done by the batch process; different amounts of resin Kyron-T-104 and Tulsion-412 were placed in beakers containing 100 mL of deionized water and allowed to swell for a definite period of time. Accurately weighed amount of Levocetrizine dihydrochloride (as per 1 : 1, 1 : 2, 1 : 3, 1 : 4, 1 : 5, 1 : 6, and 1 : 7 drug resin ratio) was added and stirred for desired period of time. The mixture was filtered and residue was washed with deionized water. Filtrate was analyzed by U.V. spectrophotometer at 231 nm for the unbound drug and percentage drug loading was calculated [26–28].

### 2.4. Formulation Development of FDTS

All formulations of FDTs were prepared by direct compression technique for batch by taking DRC equivalent to 5 mg of Levocetrizine dihydrochloride, Montelukast sodium (10 mg), MCC was used as diluent, talc as an antiadherent, and magnesium stearate as a lubricant. All the ingredients were accurately weighed and blended together to get uniform mixture. Then the blend was compressed to get tablets using rotary tablet machine. The formulations are shown in Table 1.

## 3. Result and Discussion

### 3.1. Characterizations of Drugs, DRC, and Final Blend

**Figure 1:**
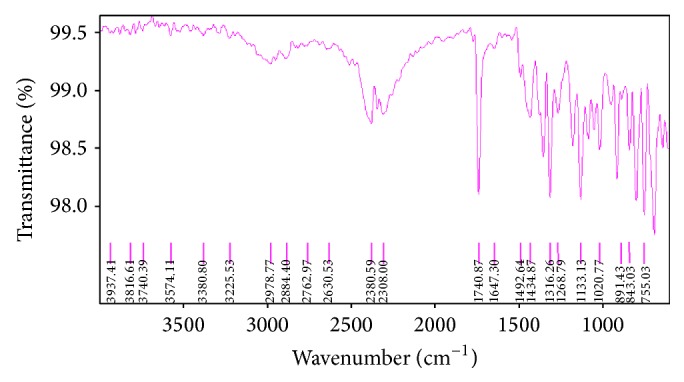
Fourier transform infrared spectra of Levocetrizine dihydrochloride.

**Figure 2:**
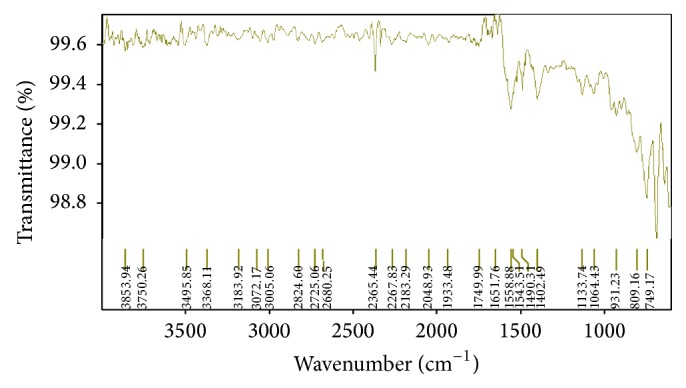
Fourier transform infrared spectra of Montelukast sodium.

**Figure 3:**
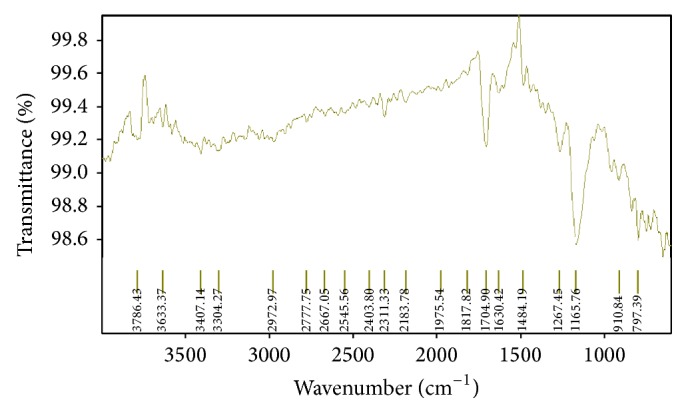
Fourier transform infrared spectra of Levocetrizine dihydrochloride and resins (Kyron-T-104, Tulsion 412).

**Figure 4:**
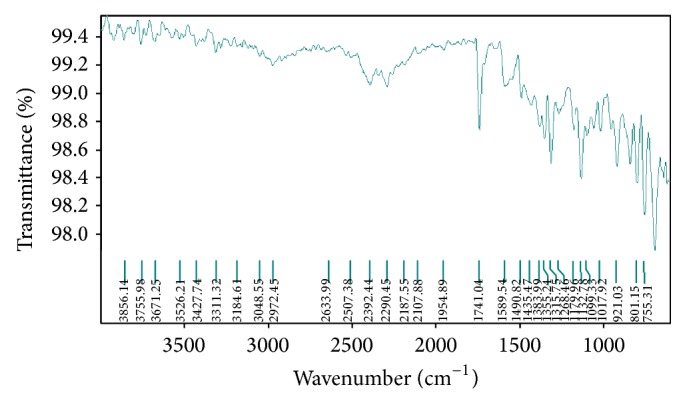
Fourier transform infrared spectra of Levocetrizine dihydrochloride, Montelukast sodium, resins, and other excipients.

The FTIR (Fourier transmission Infrared) spectroscopy study of Levocetrizine dihydrochloride, Montelukast sodium (mixed and separate), drug-resin complex, blend containing both the drug, resin, and other excipients used in the formulation development was carried out to check the compatibility to each other (Figures 1, 2, 3, and 4).

The spectra indicated that there was no drug-drug and drug-excipients interaction as the peaks of the drug and other excipients were seen the same in the drug-excipients mixture indicating that the drug molecule was present in an unchanged state in the formulation.

### 3.2. Precompression Evaluation

#### 3.2.1. Optimization of Various Conditions for Maximum Drug Loading

Drug loading process was optimized for maximum drug loading considering conditions like effect of resin activation, drug: resin ratio, pH, temperature, resin swelling time, and stirring time [18, 28–30].

*Optimization of Resin Activation*. Changing the ionic form of ion exchange resin (IER) might occasionally be required to convert a resin from one form to another, if it does not have the desired counter ions. Strongly acidic cation exchange resins are usually marketed in Na^+^ form and strongly basic anion exchange resins in Cl^−^ form. They are generally converted into hydrogen and hydroxide forms, respectively. The conversion could be achieved by soaking the resins with acid or alkali solutions, respectively. After changing the ionic form, the resin was subjected to washing with distilled water until elute becomes neutral in reaction and finally dried at 50°C. The effect of activation of resin on drug loading was studied. 100 mg of resin, placed on a Whatman filter paper in a funnel, was washed with deionized water and subsequently with 1 N HCl (100 mL). The resins were rewashed with deionized water until neutral pH was reached. DRC was prepared in the same way as discussed earlier using 100 mg each of Levocetrizine dihydrochloride and acid activated resins. Similarly, alkali activation of resin was done, replacing 1 N HCl with 1 N NaOH. Finally, Kyron T-104 and Tulsion-412 were also activated with combined treatment of 1 N HCl and 1 N NaOH solutions. Drug loading efficiency in each case was determined.

In the case of Kyron-T-104 the acid treated resin loaded maximum drug, that is, 67.24%, whereas 63.87% drug was loaded when Tulsion-412 used. The resin so activated exposed the exchangeable groups producing rapid ion exchange hence highest drug binding. Highest percentage drug loading was found for acid activated resin, but as compared to inactivated resin no major effect was found on percentage drug loading. Thus trials were made with inactivated resin. The results are shown in Table 2.

*Optimization of Drug: Resin Ratio*. 100 mg of Levocetrizine dihydrochloride was added to each of the fourteen beakers containing 100, 200, 300, 400, 500, 600, and 700 mg of resins separately swelled in 100 mL of deionized water. The mixture was stirred for 4 hrs. DRC was collected by filtration, washed with deionized water, and evaluated for drug content.

*Optimization of pH*. The study was carried out at six pH values 2, 3, 4, 5, 6, and 7. The pH was adjusted to desired value using standard solutions of HCl and NaOH. Loading efficiency was determined at these conditions.

The pH affects the extent of drug loading process. It was observed that optimum drug loading was achieved at pH 5.0 and was not much increased at pH higher than this. The results are shown in Table 3.

*Optimization of Temperature*. Temperature was optimized by preparing DRC using 100 mg Levocetrizine dihydrochloride and 300 mg resins in 100 mL of deionized water and set temperature at 20°C, 30°C, 40°C, 50°C, and 60°C using temperature controlled magnetic stirrer.

The Efficient drug loading on Kyron T-104 and Tulsion-412 occurred uniformly in the experimental temperature 30°C and the effect of temperature on drug loading is shown in Table 4.

*Optimization of Resin Swelling Time*. Optimization of resins swelling time was carried out by keeping 400 mg of resin Kyron T-104 and 500 mg of resin Tulsion-412 in each of the beakers containing 100 mL of deionized waterfor 30, 60, 90, and 120 min, respectively, on magnetic stirrer. DRC was prepared as described above using 100 mg of Levocetrizine dihydrochloride and percent drug loading was estimated.

It was noted that the resin requires proper swelling time for maximum drug loading. Swelling and hydration increase the rate and extent of ion exchange process. In unswollen resin matrix, the exchangeable groups are latent and coiled towards the backbone. Swelling increases the surface area and these groups get oriented towards outside. Loading that was considerably increased at 90 minutes was considered as the optimum swelling time. The effect of swelling time on drug loading is shown in Table 5.

*Optimization of Resin Stirring Time*. For optimizing stirring time, DRC was prepared by stirring 100 mg of Levocetrizine dihydrochloride with 400 mg of resin Kyron T-104 and 500 mg of resin Tulsion-412 in 100 mL of deionized water separately for 60, 90, 120, 180, 240, and 300 min and percent drug loading was evaluated.

Stirring time affects the ion exchange equilibrium process as it is stoichiometric process. This may indicate the significant involvement of Van-der Waals forces or chemisorptions taking place along with drug exchange during complexation. Loading was not considerably increased after 240 minutes so it was considered as the optimum contact time between Levocetrizine dihydrochloride and Kyron T-104 and Tulsion-412. The effect of stirring time on drug loading is shown in Table 6.

#### 3.2.2. Evaluation of Taste of Resinate

Taste of resinate was checked by time intensity method. For this purpose human volunteers were selected. In this method a sample equivalent to a normal dose was held in mouth for 10 seconds and volunteers were asked to evaluate the taste of resinate. Bitterness levels were recorded immediately according Strong Biter, Moderate Bitter, Slight Bitter, and Tasteless. These volunteers were instructed not to swallow resinate, which were placed on the tongue. They were instructed to thoroughly gargle their mouth with distilled water after the completion of test [31].

Optimization of drug: resin ratio had shown that complete taste masking was achieved in ratio 1 : 4 to 1 : 7 in case of both the resins. The scale of bitterness is represented in Table 7.

Optimization of drug: resin ratio was done by taking inactivated resins in ratio 1 : 1 to 1 : 7 with drug (Levocetrizine dihydrochloride). Maximum drug loading was found in ratios 1 : 4 (kyron-T-104) and 1 : 5 (Tulsion-412) so further optimizations was done with this ratio. The effect of drug-resin ratio on drug loading and relation between drug-resin and drug loading is represented in Tables 8 and 9.

#### 3.2.3. Micromeritic Properties

Prior to compression, the blend was evaluated for their micromeritic properties such as angle of repose, bulk density, tapped density, compressibility index, and Hausner's ratio.

Angle of repose was determined by fixed funnel method (static method) the powder was poured in the funnel and the circumference of powder pile was drawn with a pencil on the graph paper and the radius of base of a pile was measured at five different points and average was taken for calculating angle of repose.

Both bulk and tapped density are determined in USP specification density apparatus by pouring the blend into a graduated cylinder via a large funnel and measure the volume and weight and tapped density was measured by operating the instrument for a fixed number of taps until powder has reached a minimum volume.

Hausner's ratio indicates the flow ability and packing ability. When Hausner's ratio is close to 1, materials have acceptable flow and packing ability.

From the results of precompression studies of the batch K1–K6, it was concluded that powder mixtures has good flow and compressibility property. The bulk density of powder mixtures was found in the range of 0.438–0.465 g/cm^3^. The values of Carr's index were in the range of 12.89–13.49 and Hausner's ratio was in the range of 1.142–1.156 suggested that blend having fairly good flow. The results of precompression evaluation are shown in Table 10.

### 3.3. Postcompression Evaluation

The weight variation test was carried out in order to ensure uniformity in the weight of tablets in a batch. The total weight of randomly selected 20 tablets was determined and the average was calculated. The individual weight of the tablets was also determined accurately and the weight variation was calculated.

The permissible limit for hardness is 3–12 kg/cm^2^. The hardness test was performed by using Pfizer hardness tester.

The thickness and diameter of the tablets were determined by using vernier calipers. Randomly 10 tablets selected were used for determination of thickness that expressed in mean ± SD and unit is millimeter (mm).

The pharmacopoeia limit of friability is 1% and friability was measured using a Roche friability apparatus, carried out at 25 rpm for 4 min (100 rotations). However, it becomes a great challenge for a formulator to achieve friability within this limit for MDT product keeping hardness at its lowest possible level in order to achieve a minimum possible disintegration time. The friability (*F*%) is given by the following formula:

(1) $$\begin{array}{c} F\%=\left(1-\frac{W_{0}}{W}\right)\times 100, \end{array}$$

where *W*_0_ is weight of the tablets before the test and *W* is the weight of the tablets after test.

Tablets prepared by direct compression method were found to be good without any chipping, capping, and sticking. Various physical parameters like thickness, hardness, weight variation, friability, hardness, and disintegration time were measured to evaluate tablets. It was found that the average thickness of the tablets also ranged between 3.11 and 3.13 mm; however, the variations were not alarming and remained within the acceptable range. Hardness of tablets of the different formulations varied widely ranging from 3.0 to 3.4 kg/cm^2^. The loss in friability was ranged from 0.37 to 0.57% so all the postcompression parameters were in the limit and results are shown in Table 11 [32–36].

#### 3.3.1. Wetting Time

The wetting time of the tablets was measured using a simple procedure. Five circular tissue papers of 10 cm diameter were placed in a Petri dish containing 0.2% w/v solution of amaranth (10 mL). One tablet was carefully placed on the surface of the tissue paper. The time required to develop blue color due to amaranth water soluble dye on the upper surface of the tablets was noted as the wetting time [37].

#### 3.3.2. Water Absorption Ratio

**Figure 5:**
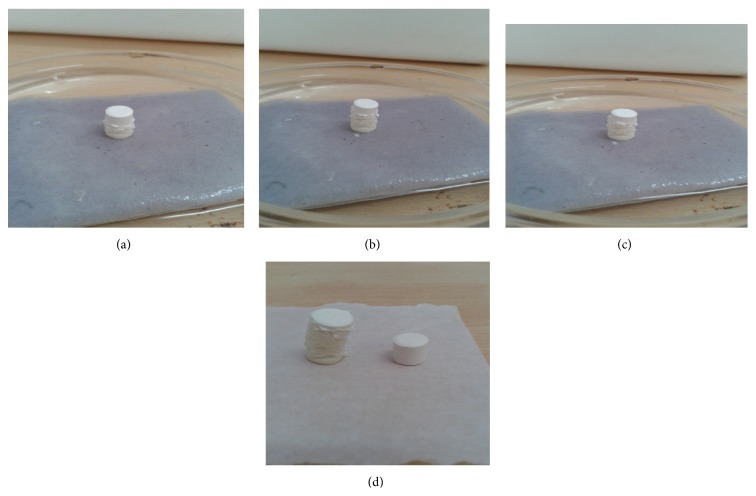
Water absorption by fast disintegrating combination tablets.

A small piece of tissue paper folded twice was placed in a small Petri dish containing 6 mL of water. A tablet was put on the paper for water absorption (Figure 5).

The wetted tablet was then weighed. Water absorption ratio, *R*, was determined by using following formula:

(2) $$\begin{array}{c} R=\frac{\left(W_{a}-W_{b}\right)}{W_{b}}\times 100. \end{array}$$

Here, *R* is the water absorption ratio, *W*_*b*_ is the weight of tablet before water absorption, and *W*_*a*_ is the weight of tablet after water absorption [38].

The water absorption ratio was found to be in range from 83.38 to 93.18%, whereas the wetting time of batch K1 to K6 was found from 19.49 to 28.32 and wetting time was significantly lower in K4 due to highly water absorption capacity (Table 12).

#### 3.3.3. Drug Content

Ten tablets were powdered and 10 mg drug equivalent powder dispersed in phosphate buffer pH 6.8. Volume of the solution made up to 10 mL by media. The mixture was filtered and 1 mL of the filtrate was diluted to 10 mL using phosphate buffer pH 6.8. The absorbance of the sample preparations was measured at *λ*_max⁡_ 231.0 nm for Levocetrizine dihydrochloride and 352.20 nm for Montelukast sodium.

Another method (Petri dish method) was used to calculate drug content in which 10 mL phosphate buffer pH 6.8 was taken in Petri dish and then a tablet was dipped in it and after 30 sec media was filtered (process repeated at least for 3 times) and the absorbance of the sample preparations was measured at *λ*_max⁡_ 231.0 nm for Levocetrizine dihydrochloride and 352.20 nm for Montelukast sodium [39].

*Uniformity of Drug Content*. Ten tablets were selected randomly and average weight was calculated for both Levocetrizine dihydrochloride and Montelukast sodium. Tablets were crushed in a mortar and accurately weighed amount of drug was taken from the crushed blend. Then, the samples were transferred to 100 mL volumetric flasks and diluted up to the mark with methanol. The content was shaken periodically and kept for one hour to dissolve the drug completely. The mixtures were filtered and appropriate dilutions were made separately for both drugs. The drug content in each tablet was estimated at *λ*_max⁡_ against blank reference and reported.

In the case of Levocetrizine dihydrochloride the drug content was found in the range of 94.65–98.74%, whereas 92.98–95.78% drug was found in case of Montelukast sodium (Table 13).

#### 3.3.4. Disintegration Time

*By Disintegration Test Apparatus*. Disintegration time is considered to be one of the important criteria in selection the best formulation. To achieve correlation between disintegration times* in vitro* and* in vivo*, several methods were proposed, developed, and followed at their convenience [40–43]. One tablet was placed into each tube and the assembly was suspended into the 1000 mL beaker containing phosphate buffer pH 6.8 maintained at 37°C. The apparatus was operated and time was taken as disintegration time when no particle of tablet remains on the mesh when it is at up position. The assembly was removed from the liquid and the tablets were observed.

*Disintegration Time in the Oral Cavity*. The healthy volunteers of either sex (age 18–25) were selected, trained, and then DT of each tablet for complete disintegration in the mouth was measured. The time when the tablet placed on the tongue disintegrated without leaving any lumps was taken as end point. After disintegration of tablet in the oral cavity the tablet contents were spit out and the oral cavity was rinsed with water. DT of six tablets per batch (three tablets per trained volunteer or total of two volunteers per batch) was recorded and the average was reported.

**Figure 6:**
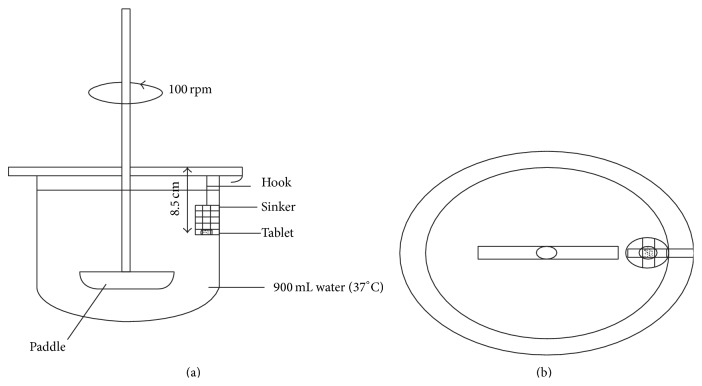
Schematic view of modified dissolution apparatus for disintegration test (from reference).

**Figure 7:**
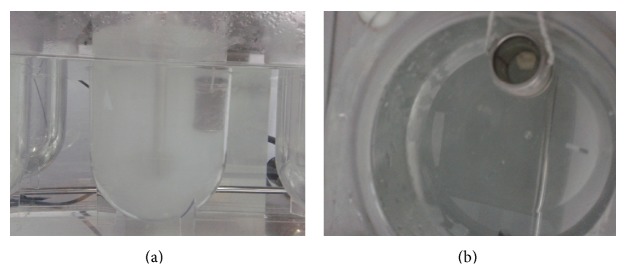
Experimental view of modified dissolution apparatus for disintegration test.

*Disintegration Test Using Modified Dissolution Apparatus*. Bi et al. suggested the use of a modified dissolution apparatus for disintegration (Figure 6), instead of the traditional disintegration apparatus. In this experiment, 900 mL of phosphate buffer (pH 6.8) was maintained at 37°C as the disintegration fluid and a paddle at 100 rpm as stirring element was used. Disintegration time was noted when the tablet disintegrated and passed completely through the screen of the sinker (3–3.5 mm in height and 3.5–4 mm in width, immersed at a depth of 8.5 cm from the top with the help of a hook). This method was useful in providing discrimination among batches which was not possible with the conventional disintegration apparatus (Figure 7).

**Figure 8:**
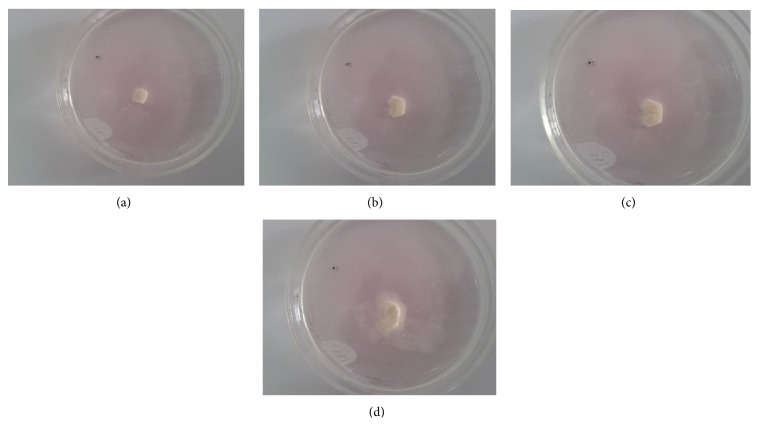
Experimental view of modified disintegration test (Petri dish method).

*Disintegration by Petri Dish Method*. Petri dish method was used to calculate drug content in which 10 mL phosphate buffer pH 6.8 was taken in Petri dish then a tablet was dipped in it (Figure 8). Disintegration time was noted when the tablet disintegrated (process repeated at least for 3 times).

As per the pharmacopoeia requirement, formulation of fast disintegrating tablet should exhibit disintegration time in ≤60 seconds; K1 to K6 batches pass the disintegration time requirement. From the above it is observed that all the prepared formulations batches exhibited disintegration time less than 60 seconds and out of these K4 and K5 batch exhibited the least disintegration time (Table 14).

In all observations K4 and K5 were found suitable for further dissolution study as an optimized batch.

### 3.4. *In Vitro* Drug Release Study

K4 and K5 batch formulations were selected for drug release study. The Levocetrizine dihydrochloride and Montelukast sodium releases from different FDTs were evaluated by using the USP30 NF25 pharmacopoeia dissolution apparatus II—paddle at 37 ± 0.5°C using 900 mL of phosphate buffer (pH 6.8) as a dissolution medium with stirring speed of 50 rpm. Aliquots (5 mL) withdrawn at various time intervals were immediately filtered through Whatman filter paper, diluted suitably and analyzed at *λ*_max⁡_ 231.0 nm for Levocetrizine dihydrochloride and 352.20 nm for Montelukast sodium [44].

The highest drug release was obtained with the formulation K4 containing super disintegrants SSG + CP in ratio of 2% (Levocetrizine dihydrochloride: Kyron-t-104 is 1 : 4). Hence, batch K4 was selected as optimized batch.

**Figure 9:**
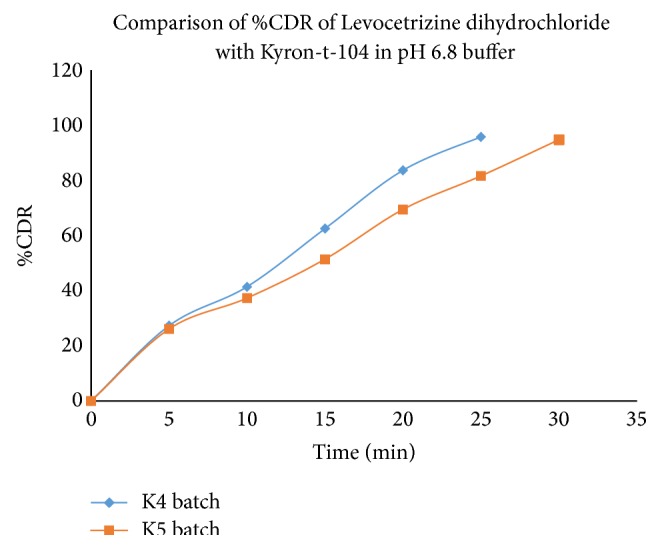
Comparison of* in vitro* drug release study of Levocetrizine dihydrochloride in K4 and K5 batch in phosphate buffer (pH 6.8).

**Figure 10:**
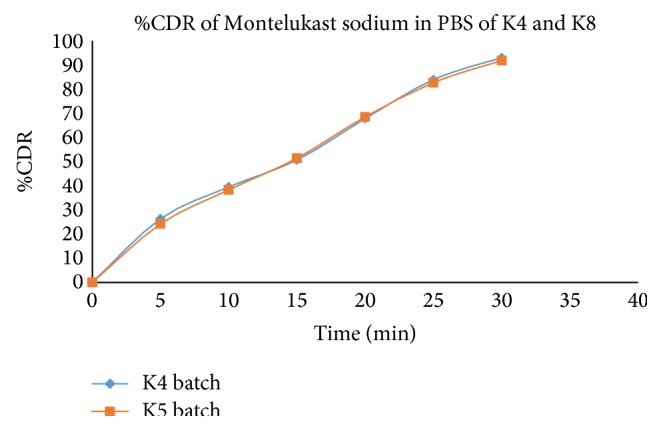
Comparison of* in vitro* drug release study of Montelukast sodium in K4 and K5 batch in phosphate buffer (pH 6.8).

The results of dissolution are shown in Tables 15 and 16 and Figures 9 and 10.

### 3.5. Stability Studies of Optimized Batch

The optimized formulations (K4 and K5) were stored in aluminum capped clear glass vials and were subjected to a storage condition of 40°C ± 2°C/75 ± 5% RH for 3 months in humidity chamber. The samples were withdrawn at time intervals of 0, 1, 2, and 3 months and evaluated for hardness, friability, dispersion time, disintegration time, drug content, and* in vitro* dissolution study [45].

**Figure 11:**
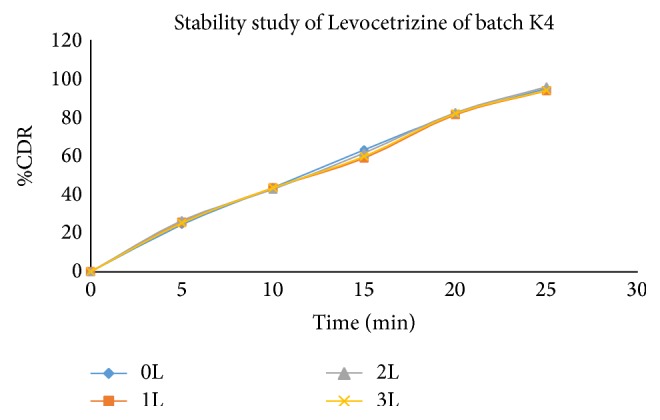
Comparative* in vitro* drug release profile of stability study batches of Levocetrizine dihydrochloride (K4 batch) in phosphate buffer (pH 6.8).

**Figure 12:**
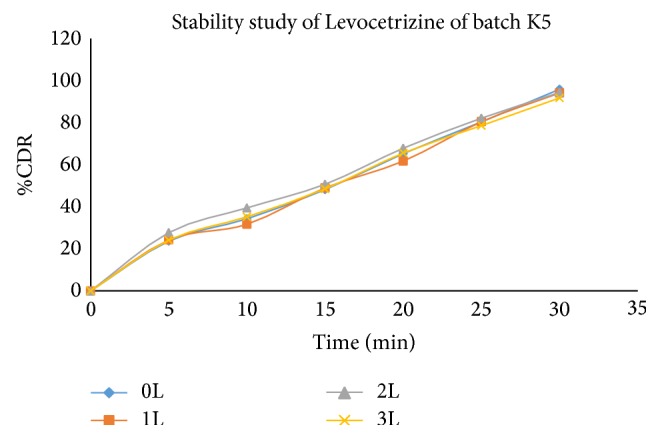
Comparative* in vitro* drug release profile of stability study batches of Levocetrizine dihydrochloride (K5 batch) in phosphate buffer (pH6.8).

**Figure 13:**
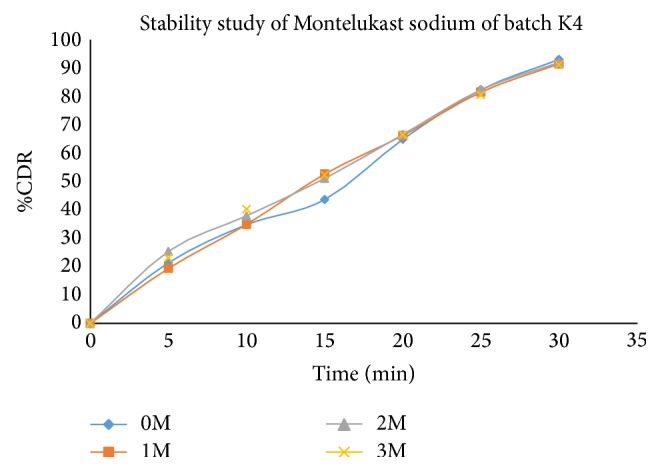
Comparative* in vitro* drug release profile of stability study batches of Montelukast sodium (K4 batch) in phosphate buffer (pH6.8).

**Figure 14:**
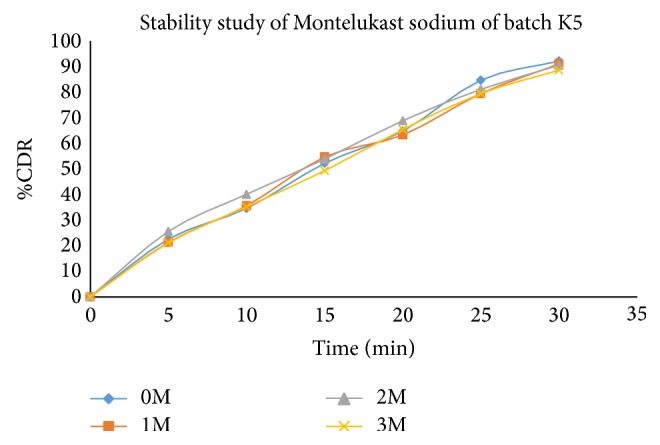
Comparative* in vitro* drug release profile of stability study batches of Montelukast sodium (K5 batch) in phosphate buffer (pH 6.8).

Stability study revealed (Tables 17, 18, and 19) that all the formulations were physically stable when stored at 40 ± 20°C and 75 ± 5% RH till 3 months and there was no significant difference in dissolution for optimized formulation (Figures 11, 12, 13, and 14).

### 3.6. Determination of Similarity and Dissimilarity Factors

A model independent approach was used to estimate dissimilarity factor (*f*_1_) and a similarity factor (*f*_2_) to compare dissolution profile of optimized calculated FDTs with FDTs containing superdisintegrants. The FDA and SUPAC-IR guidelines define difference factor (*f*_1_) as the calculated percent (%) difference between the reference and test curves at each time point and are a measurement of the relative error between the two curves.

The similarity factor (*f*_2_) is given by the following equation:

(3) $$\begin{array}{c} f_{2}=50\times \log\left\{{\left[1+\left(\frac{1}{n}\right)S_{t=1}^{n}{\left(R_{t}-T_{t}\right)}^{2}\right]}^{-0.5}\times 100\right\}. \end{array}$$

The dissimilarity factor (*f*_1_) is given by the following equation:

(4) $$\begin{array}{c} f_{1}=\left\{\frac{\left[S_{t=1}^{n}\left|R_{t}-T_{t}\right|\right]}{\left[S_{t=1}^{n}R_{t}\right]}\right\}\times 100, \end{array}$$

where *n* is the number of pull points, *R*_*t*_ is the reference batch profile at time point *t*, and *T*_*t*_ is the test batch profile at the same time *t*. For* in vitro* dissolution curves to be considered similar *f*_1_ values should be in the range of 0–15, while values of *f*_2_ should lie within 50–100 [46–49].

Similarity (*f*_2_) and dissimilarityfactors (*f*_1_) for K4 and K5 are shown in Tables 20 and 21. All formulations showed (*f*_2_) value between 50 and 100 and (*f*_1_) value below 15 indicating similar release profiles of the formulations before and after stability studies.

## 4. Conclusion

In the present work an attempt was made to use ion exchange resins (Kyron-T-104 and Tulsion-412) as taste masking agents for Levocetrizine dihydrochloride. Combinations of three superdisintegrants (separately and in ratio) were used in the formulation of fast disintegrating combination tablet of Levocetrizine dihydrochloride and Montelukast sodium. The purpose was to enhance patient compliance and provide fast onset of action. Kyron T-104 and Tulsion-412 were used as ion exchange resins and it was mixed with the drug in different ratios and evaluated for the extent of complexation. Results have shown that with Kyron T-104, drug to resin ratio of 1 : 4 and with Tulsion-412, drug to resin ratio of 1 : 5 gave maximum amount of drug loading. These drug-resin complexes further evaluated for taste masking and different conditions of drug loading and after optimization Kyron T-104 resinate with drug in ratio 1 : 4 selected for formulation development on the basis of maximum drug loading and cost effectiveness. All the blends (K1, K2, K3, K4, K5, and K6) for formulation exhibited satisfactory values for angle of repose, bulk density, Tapped density, Hausner ratio, and Carr's index and shown good flow properties. All the tablets passed the weight variation test, friability test, hardness test, and % variation and were found within the pharmacopoeia limit. Drug content estimation showed that more than 90% of the drugs (Levocetrizine dihydrochloride and Montelukast sodium) was present. The dispersion produced was smooth with pleasant mouth feel and the bitter taste was totally masked in all formulations. The disintegration tests conducted on all these formulations showed that there was faster disintegration of the tablets, taking 16 to 31 seconds, which was much less than the official limit for fast disintegrating tablets (1 minutes). Minimum time for disintegration was shown by the formulations K4 and K5 so these two formulations finally selected for drug release study.* In vitro* drug release profile of tablet shown above 90% drugs (Levocetrizine dihydrochloride and Montelukast sodium) in 25–35 minutes in phosphate buffer (pH 6.8) indicating that the drug will be absorbed faster in the mouth, pharynx, and oesophagus and thus chances of enhancement the bioavailability by pregastric absorption through mouth, pharynx, and oesophagus. Stability study was conducted for 3 months. Similarity (*f*_2_) and dissimilarity factors (*f*_1_) for K4 and K5 were calculated. All formulations showed (*f*_2_) values between 50 and 100 and (*f*_1_) value below 15 indicating similar release profiles of the formulations before and after stability studies. There was no significant change in taste and color at optimized temperature. There was no significant variation in the disintegration time, hardness, friability, and* in vitro* dissolution profiles for the optimized formulations K4 and K5. On the basis of drug release K4 was the optimized formulation but as there was no significant difference between drug release profiles and other parameters of K4 and K5 and on the basis of cost effectiveness K5 may also be considered as optimized formulation.

## Figures and Tables

**Table 1:** Details of formulations (K1–K6) of batch formulation.

Batch-1						
Levocetrizine dihydrochloride : Kyron-T-104 (1 : 4)						
Ingredients	K1	K2	K3	K4	K5	K6
Montelukast sodium	10 mg	10 mg	10 mg	10 mg	10 mg	10 mg
MCC	176 mg	176 mg	176 mg	176 mg	176 mg	176 mg
SSG	4 mg	—	—	—	—	—
CCS	—	4 mg	—	—	—	—
CP	—	—	4 mg	—	—	—
SSG + CCS	—	—	—	4 mg	—	—
SSG + CP	—	—	—	—	4 mg	—
CCS + CP	—	—	—	—	—	4 mg
Mg stearate	2 mg	2 mg	2 mg	2 mg	2 mg	2 mg
Talc	3 mg	3 mg	3 mg	3 mg	3 mg	3 mg

MCC: microcrystlline cellulose; SSG: sodium starch glycolate; CCG: Crosscarmellose sodium; CP: crosspovidone.

**Table 2:** Effect of resin activation on drug loading.

Optimized ratio of drug and resin	% Drug loading by resin activation		
	Acid	Alkali	Acid-alkali
Kyron-T-104 (1 : 4)	67.24	48.32	57.63
Tulsion-412 (1 : 5)	63.87	43.19	56.84

**Table 3:** Effect of pH on drug loading.

pH	% Drug loading	
	Kyron-T-104 (1 : 4)	Tulsion-412 (1 : 5)
2	39.25	36.64
3	45.78	44.73
4	48.43	50.19
5	76.59	74.61
6	62.08	63.38
7	64.17	68.25

**Table 4:** Effect of temperature on drug loading.

Temperature (°C)	% Drug loading	
	Kyron-T-104 (1 : 4)	Tulsion-412 (1 : 5)
20	58.24	56.15
30	79.35	72.31
40	74.16	60.46
50	47.93	49.16
60	38.42	34.81

**Table 5:** Effect of swelling time on drug loading.

Swelling time (min)	% Drug loading	
	Kyron-T-104 (1 : 4)	Tulsion-412 (1 : 5)
30	71.36	74.18
60	86.38	84.42
90	88.13	87.75
180	74.69	74.18

**Table 6:** Effect of stirring time on drug loading.

Stirring time (min.)	% Drug Loading	
	Kyron-T-104 (1 : 4)	Tulsion-412 (1 : 5)
60	78.63	74.52
120	79.58	80.69
180	84.18	81.66
240	89.37	86.24
300	83.74	82.91

**Table 7:** Scale for bitterness evaluation.

Drug (Levocetrizine dihydrochloride) : resin	Kyron-T-104	Tulsion-412
	Bitterness evaluation	
1 : 1	Strong bitterness	Strong bitterness
1 : 2	Moderate to strong bitterness	Moderate to strong bitterness
1 : 3	Slightly to moderately bitter	Slightly to moderately bitter
1 : 4	Tasteless	Tasteless
1 : 5	Tasteless	Tasteless
1 : 6	Tasteless	Tasteless
1 : 7	Tasteless	Tasteless

**Table 8:** Effect of drug-resin ratio on drug loading.

Resin	% Drug loading in different ratios of drug (Levocetrizine dihydrochloride) : resin						
	1 : 1	1 : 2	1 : 3	1 : 4	1 : 5	1 : 6	1 : 7
Kyron-T-104	54.72	60.39	64.28	85.26	73.22	70.11	71.49
Tulsion-412	56.43	62.56	65.38	74.39	80.93	75.43	77.28

**Table 9:** Relation between drug-resin ratio and drug loading.

Optimized ratio of drug and resin	% Drug loading
Kyron-T-104 (1 : 4)	85.26 ± 1.32
Tulsion-412 (1 : 5)	80.93 ± 1.48

**Table 10:** Precompression evaluation of Levocetrizine dihydrochloride and Montelukast sodium blend.

Formulation batch	Precompression parameters					
	Angle of Repose	Bulk density	Tapped density	Hausner ratio	Carr's index	% compressibility
K1	34.68	0.465	0.536	1.152	13.19	13.24
K2	32.25	0.448	0.512	1.142	12.43	12.50
K3	32.72	0.453	0.524	1.156	13.49	13.54
K4	33.57	0.438	0.503	1.148	12.89	12.92
K5	34.26	0.453	0.521	1.150	13.04	13.05
K6	31.48	0.435	0.502	1.154	13.34	13.35

**Table 11:** Postcompression evaluation of developed formulations.

Formulation	Postcompression evaluation parameters				
	Thickness (mm)	Diameter (mm)	Hardness (Kg/cm^2^)	Wt. variation	Friability
K1	3.12 ± 0.17	3.0 ± 0.38	3.1 ± 0.68	219.03 ± 1.08	0.49 ± 0.082
K2	3.13 ± 0.14	3.0 ± 0.27	3.2 ± 0.73	218.59 ± 0.94	0.52 ± 0.068
K3	3.12 ± 0.19	3.1 ± 0.28	3.4 ± 0.69	219.13 ± 1.11	0.57 ± 0.072
K4	3.11 ± 0.13	3.0 ± 0.24	3.2 ± 0.47	219.44 ± 0.92	0.47 ± 0.069
K5	3.13 ± 0.11	3.0 ± 0.33	3.3 ± 0.81	220.19 ± 0.91	0.53 ± 0.036
K6	3.12 ± 0.17	3.0 ± 0.36	3.0 ± 0.71	218.31 ± 0.89	0.37 ± 0.074

**Table 12:** Determination of wetting time and % water absorption of developed formulations.

Formulation batch	Wetting time (sec.)	% Water absorption
K1	28.32 ± 0.524	88.16 ± 0.985
K2	24.41 ± 0.331	83.38 ± 0.886
K3	23.62 ± 0.632	90.43 ± 0.894
K4	19.49 ± 0.309	93.18 ± 0.734
K5	21.76 ± 0.412	90.57 ± 0.759
K6	22.13 ± 0.476	91.79 ± 0.804

**Table 13:** Determination of drug contents by different methods.

Formulation	Dispersion time (sec.)	Drug content (%) by dilution method		Drug content (%) by Petri dish method	
		Levocetrizine dihydrochloride	Montelukast	Levocetrizine dihydrochloride	Montelukast
K1	31.41 ± 0.41	94.65 ± 1.08	93.53 ± 1.15	96.02 ± 1.26	95.14 ± 1.14
K2	30.15 ± 0.53	96.38 ± 1.13	94.17 ± 1.06	95.28 ± 1.23	93.04 ± 1.19
K3	26.32 ± 0.64	96.68 ± 1.05	93.88 ± 0.98	97.41 ± 1.19	94 .53 ± 1.26
K4	19.79 ± 0.59	98.14 ± 0.97	95.63 ± 0.92	98.74 ± 1.16	95.78 ± 1.16
K5	22.62 ± 0.47	97.46 ± 1.16	94.05 ± 1.02	98.10 ± 1.24	93.92 ± 1.28
K6	24.58 ± 0.68	98.22 ± 1.14	92.98 ± 1.13	96.68 ± 1.18	95.11 ± 1.17

**Table 14:** Determination of disintegration time by different methods.

Formulation	Disintegration by disintegration apparatus (sec.)	Disintegration Time in the Oral Cavity (DT).	Disintegration by Petri dish method (sec.)	Disintegration by dissolution apparatus with basket (sec.)
K1	23 ± 0.76	24 ± 0.16	31 ± 0.89	28 ± 0.43
K2	20 ± 0.63	26 ± 0.24	32 ± 0.72	29 ± 0.51
K3	19 ± 0.58	22 ± 0.19	30 ± 0.94	26 ± 0.47
K4	16 ± 0.55	21 ± 0.25	21 ± 0.67	20 ± 0.39
K5	16 ± 0.61	22 ± 0.21	23 ± 0.74	21 ± 0.44
K6	20 ± 0.51	24 ± 0.18	26 ± 0.69	24 ± 0.53

**Table 15:** *In vitro* drug release study of K4 batch (Kyron-T-104) in phosphate buffer (pH 6.8).

Dissolution media →	Phosphate buffer pH 6.8	
Time (min) ↓	L	M
0	0	0
5	27.34	26.31
10	41.46	39.46
15	62.57	50.62
20	83.73	67.74
25	95.83	83.87
30		92.92

L: Levocetrizine dihydrochloride; M: Montelukast sodium.

**Table 16:** *In vitro* drug release study of K5 batch (Kyron-T-104) in phosphate buffer (pH 6.8).

Dissolution media →	Phosphate buffer pH 6.8	
Time (min) ↓	L	M
0	0	0
5	26.16	24.18
10	37.35	38.26
15	51.46	51.37
20	69.54	68.49
25	81.67	82.64
30	94.82	91.74

L: Levocetrizine dihydrochloride; M: Montelukast sodium.

**Table 17:** Stability study data for optimized formulation K4 and K5.

Formulation batch	Parameters evaluated	Time interval (months)							
		0		1		2		3	
K4	Hardness (kg/cm^2^)	3.1 ± 0.47		3.2 ± 0.38		3.1 ± 0.24		3.3 ± 0.42	
	Friability (%)	0.49 ± 0.089		0.51 ± 0.072		0.43 ± 0.091		0.39 ± 0.103	
	Dispersion time (sec)	20.37 ± 0.16		19.78 ± 0.21		19.64 ± 0.39		21.31 ± 0.25	
	Drug content (%)	**L**	**M**	**L**	**M**	**L**	**M**	**L**	**M**
		97.6	93.2	96.3	94.7	96.9	93.6	98.2	92.5
	Disintegration time (sec)	18 ± 0.12		20 ± 0.16		18 ± 0.07		19 ± 0.21	

K5	Hardness (kg/cm^2^)	3.3 ± 0.52		3.2 ± 0.41		3.1 ± 0.62		3.3 ± 0.57	
	Friability (%)	0.58 ± 0.076		0.63 ± 0.069		0.48 ± 0.062		0.51 ± 0.092	
	Dispersion time (sec)	23.69 ± 0.89		22.54 ± 0.73		22.79 ± 0.83		21.39 ± 51	
	Drug content (%)	**L**	**M**	**L**	**M**	**L**	**M**	**L**	**M**
		98.2	94.3	96.7	94.9	97.4	93.5	98.3	95.1
	Disintegration time (sec)	20 ± 0.06		21 ± 0.15		21 ± 0.11		19 ± 0.18	

L: Levocetrizine dihydrochloride; M: Montelukast sodium.

**Table 18:** Dissolution profile of stability study batch K4.

% Cumulative drug release in phosphate buffer (pH 6.8)—Kyron-T-104 (SSG + CP)								
Month batch →	0		1		2		3	
Time (min) ↓	L	M	L	M	L	M	L	M
5	24.43	21.38	25.62	19.38	26.51	25.43	25.23	23.10
10	43.57	34.79	43.37	34.89	42.78	37.92	43.42	40.25
15	63.13	43.62	58.82	52.66	61.48	51.07	59.68	52.31
20	81.68	64.91	81.36	66.23	82.39	66.61	81.91	66.47
25	94.86	82.41	93.71	81.56	95.72	82.39	93.86	80.54
30		93.06		91.49		91.98		91.23

L: Levocetrizine dihydrochloride; M: Montelukast sodium.

**Table 19:** Dissolution profile of stability study batch K5.

% Cumulative drug release in phosphate buffer (pH 6.8)—Kyron-T-104 (SSG + CCS)								
Month batch →	0		1		2		3	
Time (min) ↓	L	M	L	M	L	M	L	M
5	23.72	22.61	24.23	21.32	27.73	25.64	26.23	21.13
10	34.28	34.56	31.69	35.58	39.49	40.11	43.42	35.20
15	48.15	52.23	49.01	54.73	50.72	53.87	59.68	49.32
20	65.29	64.79	61.72	63.29	67.87	68.91	81.91	65.44
25	80.38	84.73	80.44	79.48	82.13	81.17	93.86	79.54
30	95.89	92.19	94.11	91.08	94.37	90.54	91.78	88.62

L: Levocetrizine dihydrochloride; M: Montelukast sodium.

**Table 20:** Similarity factor and dissimilarity factors of Levocetrizine dihydrochloride in K4 and K5 before and after stability study.

Time (min)	% Cumulative drug release of before stability study (*R*_*t*_)		% Cumulative drug release of after stability study (*T*_*t*_)		L with (SSG + CCS)		L with (SSG + CP)	
	L with (SSG + CCS)	L with (SSG + CP)	L with (SSG + CCS)	L with (SSG + CP)	*f*_1_	*f*_2_	*f*_1_	*f*_2_
5	26.16	27.34	24.18	29.23	3.64	79.60	1.98	85.90
10	37.35	41.46	35.23	43.42				
15	51.46	62.57	48.52	59.68				
20	69.54	83.73	65.58	81.91				
25	81.67	95.83	78.60	93.86				
30	94.82		91.78					

L: Levocetrizine.

**Table 21:** Similarity factor and dissimilarity factors of Montelukast sodium in K4 and K5 before and after stability study.

Time (min)	% Drug release of before stability study (*R*_*t*_)		% Drug release of after stability study (*T*_*t*_)		M with (SSG + CCS)		M with (SSG + CP)	
	M with (SSG + CCS)	M with (SSG + CP)	M with (SSG + CCS)	M with (SSG + CP)	*f*_1_	*f*_2_	*f*_1_	*f*_2_
1	24.18	26.31	21.13	23.10	4.12	77.17	3.05	82.59
2	38.26	39.46	35.20	40.25				
3	51.37	50.62	49.32	52.31				
5	68.49	67.74	65.44	66.47				
8	82.64	83.87	79.54	80.54				
12	91.74	92.92	88.62	91.23				

M: Montelukast sodium.

## References

[1] Bircan Y., Çomoğlu T. (2012). Formulation technologies of orally fast disintegrating tablets. *Marmara Pharmaceutical Journal*.

[2] Deshmukh V. N. (2012). Mouth dissolving drug delivery system: a review. *International Journal of PharmTech Research*.

[3] Prateek S., Ramdayal G., Kumar S. U., Ashwani C., Ashwini G., Mansi S. (2012). Fast dissolving tablets: a new venture in drug delivery. *American Journal of PharmTech Research*.

[4] Sharma D., Singh D. K., Singh G. M., Rathore M. S. (2012). Fast disintegrating tablets: a new era in novel drug delivery system and new market opportunities. *Journal of Drug Delivery & Therapeutics*.

[5] Ashish P., Harsoliya M. S., Pathan J. K., Shruti S. (2011). A review- formulation of mouth dissolving tablet. *International Journal of Pharmaceutical and Clinical Science*.

[6] Nagar P., Singh K., Chauhan I. (2011). Orally disintegrating tablets: formulation, preparation techniques and evaluation. *Journal of Applied Pharmaceutical Science*.

[7] Pawar P. B., Mansuk A. G., Ramteke K. H., Sharma Y. P., Patil S. N. (2011). Mouth dissolving tablet: a review. *International Journal of Herbal Drug Research*.

[8] Gupta A. K., Mittal A., Jha K. K. (2011). Fast dissolving tablet- a review. *The Pharma Innovation*.

[9] Kumar A., Bhushan V., Singh M., Chauhan A. (2011). A review on evaluation and formulation of fast dissolving tablets. *International Journal of Drug Research and Technology*.

[10] Agrawal V. A., Rajurkar R. M., Thonte S. S., Ingale R. G. (2011). Fast disintegrating tablet as a new drug delivery system: a review. *Pharmacophore*.

[11] Nayak A. K., Manna K. (2011). Current developments in orally disintegrating tablet technology. *Journal of Pharmaceutical Education and Research*.

[12] Ölmez S. S., Vural İ. (2011). Advantages and quality control of orally disintegrating tablets. *Journal of Pharmaceutical Sciences*.

[13] Jagani H., Patel R., Upadhyay P., Bhangale J., Kosalge S. (2011). Fast dissolving tablets: present and future prospectus. *Journal of Advances in Pharmacy and Healthcare Research*.

[14] Gupta A., Mishra A. K., Gupta V., Bansal P., Singh R., Singh A. K. (2010). Recent trends of fast dissolving tablet—an overview of formulation technology. *International Journal of Pharmaceutical & Biological Archives*.

[15] Datrange P., Kulkarni S., Padalkar R. R. (2012). Development of taste masked formulation for bitter drug. *Research Journal of Pharmaceutical Biological and Chemical Sciences*.

[16] Wadhwa J., Puri S. (2011). Taste masking: a novel approach for bitter and obnoxious drugs. *International Journal of Biopharmaceutical & Toxicological Research*.

[17] Puttewar T. Y., Kshirsagar M. D., Chandewar A. V., Chikhale R. V. (2010). Formulation and evaluation of orodispersible tablet of taste masked doxylamine succinate using ion exchange resin. *Journal of King Saud University: Science*.

[18] Sharma V., Chopra H. (2012). Formulation and evaluation of taste masked mouth dissolving tablets of levocetirizine hydrochloride. *Iranian Journal of Pharmaceutical Research*.

[19] Dekivadia M., Gudigennavar A., Patil C., Umarji B. (2012). Development & optimization of fast dissolving tablet of levocetirizine HCl. *International Journal of Drug Development & Research*.

[20] Kanungo S., Ali S. M. A., Samanta A., Alli S. M. A. (2011). Crosslink polyacrilic resin based levocetirizine melt-in-mouth tablets. *International Journal of Pharma Sciences and Research*.

[21] Moiz M., Srinivas M. P., Sadanandam M. (2011). Formulation and evaluation of bilayered tablets of montelukast and levocetrizinedihydrochloride using natural and synthetic polymers. *International Journal of Drug Delivery*.

[22] Reddy S., Gonugunta C. S., Veerareddy P. R. (2009). Formulation and evaluation of taste-masked levocetirizine dihydrochloride orally disintegrating tablets. *Journal of Pharmaceutical Science and Technology*.

[23] Madhuri P., Srikrishna S., Kumar M. S., Subramanyam K. V., Matsyagiri L. (2012). Formulation and evaluation of sublingual tablets of an antiasthmatic drug. *International Journal of Pharma World Research*.

[24] Mahesh E., Kumar G. B. K., Ahmed M. G., kumar K. (2012). Formulation and evlaution of montelukast sodium fast dissolving tablets. *Asian Journal of Biomedical and Pharmaceutical Sciences*.

[25] Sivakranth M., Althaf A. S., Rajasekhar S. (2011). Formulation and evaluation of oral fast dissolving tablets of sildenafil citrarte. *International Journal of Pharmacy and Pharmaceutical Sciences*.

[26] Gandhi G. S., Mundhada D. R., Bhaskaran S. (2011). Levocetirizine orodispersible tablet by direct compression method. *Journal of Applied Pharmaceutical Science*.

[27] Shilpa S. K., Kumar M. A., Garigeyi P. (2011). Formulation and optimization of clopidogrel bisulfate immediate release tablet. *International Journal of Pharmaceutical Frontier Research*.

[28] Kadliya P. N., Chauhan K. V., Patel K. N., Patel P. A. (2013). Comparison and evaluation of bitter taste masked levocetrizine di hydrochloride using *β*-cyclodextrin and kyron T-114. *International Journal for Pharmaceutical Research Scholars*.

[29] Ghuge N. M., Bhople A., Thakre A. R. (2012). Formulation and evaluation of taste masked fast dissolving tablets of risperidone by using kyron T-104. *Indo American Journal of Pharmaceutical Research*.

[30] Gupta M. M., Patel V. (2013). Formulation and evaluation oral dispersible tablet of cinnarizine. *Journal of Drug Delivery & Therapeutics*.

[31] Garg A., Gupta M. M. (2013). Taste masking and formulation development & evaluation of mouth dissolving tablets of levocetrizine hydrochloride. *Journal of Drug Delivery & Therapeutics*.

[32] Chauhan B. S., Chauhan L. S., Chatterjee A., Niraj G., Pandey S., Baghel U. S. (2013). An approach based on advantages over conventional system. *Journal of Biomedical and Pharmaceutical Research*.

[33] Saroha K., Kumar G., Paul Y. (2013). Formulation and evaluation of fast dissolving tablets of amoxicillin trihydrate using synthetic superdisintegrants. *International Journal of Pharma and Bio Sciences*.

[34] Dhiman S., Verma S. (2012). Optimization of melt-in-mouth tablets of levocetirizine dihydrochloride using response surface methodology. *International Journal of Pharmacy and Pharmaceutical Sciences*.

[35] Modi A., Pandey A., Singh V., Bonde C. G., Jain D., Shinde S. (2012). Formulation and evaluation of fast dissolving tablets of diclofenac sodium using different superdisintegrants by direct compression method. *Pharmacia*.

[36] Kulkarni U., Rao N. G. R. (2011). Design and development of aceclofenac fast dissolving tablets by amorphous solid dispersion technique using modified aeglemarmelos gum. *International Journal of Pharmaceutical Research and Development*.

[37] Nayak R. K., Swamy V. B. N., Senthil A., Hardikkumar T., Kumar D. M., Mahalaxmi R. (2011). Formulation and evaluation of fast dissolving tablets of lornoxicam. *Pharmacologyonline*.

[38] Kumari S., Visht S., Sharma P. K. (2010). Preparation and evaluation of fast disintegrating tablets of dicyclomine hydrochloride. *Scholars Research Library Der Pharmacia Lettre*.

[39] Mohanachandran P. S., Mohan P. R. K., Saju F., Bini K. B., Babu B., Shalina K. K. (2010). Formulation and evaluation of mouth dispersible tablets of amlodipine besylate. *International Journal of Applied Pharmaceutics*.

[40] Kannuri R., Challa T., Chamarthi H. (2011). Taste masking and evaluation methods for orodispersible tablets. *International Journal of Pharmacy & Industrial Research*.

[41] Lalji V., Gupta M. M. (2013). Oral disintegrating tablet of antihypertensive drug. *Journal of Drug Delivery & Therapeutics*.

[42] Gandhi G. S., Mundhada D. R., Bhaskaran S. (2011). Levocetirizine orodispersible tablet by direct compression method. *Journal of Applied Pharmaceutical Science*.

[43] Shukla D., Chakraborty S., Singh S., Mishra B. (2009). Mouth dissolving tablets: an overview of evaluation techniques. *Scientia Pharmaceutica*.

[44] Kavitha K., Sandeep D. S., Yadawad M., Mangesh M. (2011). Formulation and evaluation of oral fast dissolving tablets of promethazine HCL by sublimation method. *International Journal of PharmTech Research*.

[45] Rao N. G. R., Venkatesh K., Kulkarni U., Reddy M. S., Kistayya C. (2012). Design and development of fast dissolving tablets containing baclofen by direct compression method. *International Journal of Pharmaceutical and Biomedical Research*.

[46] Moore J. W., Flanner H. H. (1996). Mathematical comparison of dissolution profiles. *Pharmaceutical Technology*.

[47] Gupta M. M., Kedawat M., Maharaj S. (2014). Reproducibility determination of optimized batches of aceclofenac and quetiapinefumarate spherical crystals by comparing the dissolution profile employing the similarity and dissimilar factors. *International Journal of Pharmaceutical Research and Development*.

[48] Goel H., Vora N., Rana V. (2008). A novel approach to optimize and formulate fast disintegrating tablets for nausea and vomiting. *AAPS PharmSciTech*.

[49] Patel N., Chotai N., Patel J., Soni T., Desai J., Patel R. (2008). Comparison of in vitro dissolution profiles of oxcarbazepine-HP *β*-CD tablet formulations with marketed oxcarbazepine tablets. *Dissolution Technologies*.

